# Potential impact of gut *Lactobacillus acidophilus* and *Bifidobacterium bifidum* on hepatic histopathological changes in non-cirrhotic hepatitis C virus patients with different viral load

**DOI:** 10.1186/s13099-022-00501-4

**Published:** 2022-06-15

**Authors:** Zeinab Ashour, Rasha Shahin, Zeinab Ali-Eldin, Mohamed El-Shayeb, Tarek El-Tayeb, Salwa Bakr

**Affiliations:** 1 Department of Internal Medicine, Faculty of Medicine, Ain Shams University, Cairo, Egypt; 2 Department of Agricultural Microbiology, Faculty of Agriculture, Ain Shams University, Cairo, Egypt; 3 Department of Clinical Pathology/Hematology & Transfusion Medicine, Faculty of Medicine, Fayoum University, Fayoum, 63514 Egypt

**Keywords:** HCV, Hepatic fibrosis, *Lactobacillus acidophilus*, *Bifidobacterium bifidum*, Lactic acid bacteria

## Abstract

**Background:**

Composition of gut microbiota has recently been suggested as a key factor persuading the pathogenesis of numerous human diseases including hepatic cirrhosis*.*

**Objective:**

To evaluate the potential impact of *Lactobacillus acidophilus* and *Bifidobacterium bifidum* microbiota on the progression of hepatic histopathological changes among patients with non-cirrhotic chronic hepatitis C (HCV) infection with different viral load. Additionally, to assess fecal composition of *Lactobacillus acidophilus ATCC-4356* and *Bifidobacterium bifidum ATCC-11863* microbiota genotypes

**Material and methods:**

This study was carried out on 40 non-cirrhotic chronically infected HCV patients, and 10 healthy-controls. Liver biopsy and HCV genomic viral load were assessed for all patients after full clinical examination. *Lactobacillus acidophilus ATCC-4356* and *Bifidobacterium bifidum ATCC-11863* microbiota were assessed in all fecal samples using PCR assay, after counting total lactic acid bacteria.

**Results:**

There was a significantly higher difference between the count of both total lactic acid and *Lactobacillus acidophilus* of healthy controls compared to patients (P-value < 0.001). Though the count of total lactic acid bacteria, and *Lactobacillus acidophilus* were higher in the cases with early stage of fibrosis (score ≤ 1) compared to those with score > 1, there were no statistically significant differences with both the serum level of hepatitis C viremia (P = 0.850 and 0.977 respectively) and the score of fibrosis (P = 0.246 and 0.260 respectively). Genotypic analysis for the composition of the studied microbiota revealed that diversity was higher in healthy controls compared to patients.

**Conclusions:**

The progression of hepatic fibrosis in HCV chronically infected patients seems to be plausible based on finding the altered *Lactobacillus acidophilus* and *Bifidobacterium bifidum* gut microbiota composition. Thus, modulation of these microbiota seems to be a promising target for prevention and control of HCV infection.

## Introduction

Liver disease is a major health problem worldwide that accounts for high morbidity and mortality with approximately two million deaths annually mainly due to complications of cirrhosis [1, 2]. Hepatitis C virus (HCV) has been evaluated to infect 130–170 million people worldwide [3]. In Egypt, the estimated prevalence of HCV is highest at > 10% of the general population [4]. Lehman et al. reported, that the epidemic of HCV infection may lead to an actual health problem with subsequent economic burden over the next ten to twenty years [5].

In the majority of patients with HCV infection, neither the early innate nor the later adaptive immune response is able to clear the virus successfully, hence, infection turns into chronic. Moreover, in a lot of cases, the infection remains undiagnosed with the persistence of ineffective inflammatory response that drives fibrogenesis with subsequent development of liver cirrhosis [6]. Therefore, understanding the underlying immune pathology is necessary for controlling the disease progression, if preventative and curative therapies are to be developed [7]. It was reported that intestinal microbiota plays a role in the pathogenesis of chronic hepatic disease [8], because the gut-liver anatomical and functional connection through hepatic portal venous system [9]. It was estimated that 10–100 trillion microorganisms reside in adult human gut from 300 to 500 various species worldwide [1]. The composition of adult gut microbiota differs widely between individuals depending on many factors; such as host genetics, diet intake, medication, as well as, other environmental factors [10]. Several diseases can alter the gut community, as well, such as; colorectal cancer, rheumatoid arthritis, anxiety, depression, autism, obesity and others [8].

Probiotics are one of the living microbiota preparation that act inside the gastrointestinal tract and have a positive impact on health [1]. *Lactobacilli* and *Bifidobacteria,* are the main genera of these probiotics [11, 12], which are known to be effective immunomodulators. *Lactobacillus acidophilus* which is one of the most important, as well as, frequent intestinal flora and may change rapidly than the changes in bowel conditions, was found to increase the cytotoxic activity of natural killer cells [13]. In previous studies, composition of gut microbiota was suggested to play a crucial role in the cross-talk of the gut-liver axis through inhibition of inflammation, suppression of oxidative stress, and prevention of hepatic deposition of lipid [1]. In another experimental study, administration of the probiotic supplementation in rats exhibited the ability of ameliorating liver pathology, decreasing liver aminotransferase and glycometabolic biomarkers, restoring intestinal barrier integrity, modulating ameliorating gut microbiota dysbiosis reducing serum inflammatory cytokines, possibly through triggering of the lipopolysaccharide/toll-like receptor 4 (LPS/TLR4) signaling pathway, which consequently activate cellular immune responses [8, 14]. Therefore, it was reported that probiotic therapies might be a promising target to prevent and control liver disease [1].

Alyet al. suggested in their study on Egyptian patients with stage 4 chronic liver disease that chronic hepatitis C can be remodeled by gut microbiome [8]. Moreover, Heidrich et al. demonstrated on their investigation for the microbial patterns in patients with different stages of chronic hepatitis C, that HCV infection was associated with variable microbial patterns even in the absence of advanced hepatic fibrosis or cirrhosis [15]. Furthermore, the cirrhosis dysbiosis ratio (CDR) was suggested as a useful semi-quantitative index to describe microbiome alteration accompanying cirrhosis progression [16]. Hence, further studies was recommended to investigate the stool microbiome profile in patients with chronic liver disease [8].

Although the previous studies suggestion that microbial translocation might be associated with the degree of liver disease in patients with HCV infection [8], a direct relation between viral hepatitis and bacterial translocation has not been established yet. Because little is known about the progress of fibrosis in viral C hepatitis and lactic acid gut microbiota, this study aimed to evaluate the potential impact of lactic acid bacteria particularly *Lactobacillus acidophilus* and *Bifidobacterium bifidum* gut microbiota on the progression of hepatic histopathological changes among patients with non-cirrhotic chronic hepatitis C (HCV) infection with different viral load. Additionally, to assess fecal composition of *Lactobacillus acidophilus ATCC-4356* and *Bifidobacterium bifidum ATCC-11863* microbiota genotypes.

## Material and methods

### Patients population

This case control study was conducted on 40 non-cirrhotic chronically infected patients with hepatitis C virus (group I), and 10 healthy control subjects well matched with respect to age and sex (group II) after obtaining their informed consent. This study was carried out in Hepatology outpatient clinic, Internal Medicine department, Ain Shams University Hospital after taking the approval of the Research Ethical Committee of the institute. Our exclusion criteria included all patients complaining of chronic hepatitis other than HCV, bilharziasis, splenomegaly, ascites, portal hypertension, recent diarrhea or constipation, liver cirrhosis, collagen disease, diabetes mellitus, or other medical illness, and any patient on alcohol intake, antibiotic, interferon or any antiviral medication. Detailed history taking, clinical examination, abdominal ultrasound (U/S), and laboratory investigations including complete blood count (CBC), liver function tests, serum transaminases, Hepatitis B surface antigen (HBs Ag), and Human immune deficiency antibody (HIV Ab) were done for both groups.

### Quantitative assessment of the RNA viral load

HCV-RNA level was detected using real-time polymerase chain reaction (RT-PCR) (Bioline International, UK) with a lower limit of detection 15 IU/ml. Mild viremia considered when the detected copy number of the virus < 200,00 IU/ml. Moderate viremia ranged from 200,000 to 2000,000 IU/ml. High viremia considered when the viral load more than 2,000,000 IU/ml [17].

### Liver biopsy and histopathology

Percutaneous liver biopsy was exclusively performed for the group I under U/S guidance using 16 gauge needles. The fixation of tissue samples were done in buffered formalin for 2–4 hrs then embedded in paraffin with melting point 55–57 °C. Sections of 3–4 um were cut followed by staining with haematoxylin-eosin and Masson's trichrome stains to identify collagen fibers. Specimens of 2.5 cm in length, including at least of 12 portal tracts were considered efficient for adequate grading and staging. They were scored for overall necro-inflammatory activity (grades 0–3) and fibrosis (stages 0–4) according to the Metavir scoring system. Examination of liver biopsies was done by only one pathologist who was blind to the clinical data.

### Detection of total lactic acid bacteria and Lactobacillus acidophilus using stool analysis and culture

De Man Rogosa Sharp (MRS) agar (CONDA, Spain) was used for the culture of lactic acid bacteria from fecal samples that were collected in the early morning. For quantitative culture of *Lactobacillus acidophilus*, fecal samples were diluted in sterile saline (weighing 1 gm in 2 ml saline). Sequential dilutions in the sterile saline solution were prepared followed by inoculation of 1 ml of each dilution into MRS agar plates using a standard loop. Then, the plates were incubated anaerobically at 37 °C for 2–3 days. For each stool specimen, colony forming unit per gram was calculated using the following formula (CFU/gm) = (D × N × 2)/W, where D: the dilution; N: the number of colonies on the plate; **2:** the original dilution of fecal specimen; and W: the weight of fecal specimen in gm). Normal bacterial count reaches up to 100,000 CFU/ml.

*Lactobacillus acidophilus* was identified using the morphology of colonies. Typical colonies of *Lactobacillus acidophilus* on MRS agar appear as small, round, rough, white, translucent, raised colonies and catalase negative. Under microscope *Lactobacillus acidophilus* appears as gram positive bacilli or rods arranged in short chains. For distinguishing *Lactobacillus acidophilus* from other lactobacilli, biochemical reactions were performed. As, *Lactobacillus acidophilus* shows inability to ferment lactose, mannitol and sorbitol [18]. The confirmatory polymerase chain reaction (PCR) assay, which was subjected on every single colony, was used as a specific identifier to the lactic acid bacteria species; (*Lactobacillus acidophilus ATCC 4356* and *Bifidobacterium bifidum ATCC 11863* microbiota).

### PCR detection of the lactic acid bacteria in fecal samples

#### DNA extraction

QIA amp DNA stool mini kit (QIAGEN, Hilden, Germany) was used for genomic isolation of bacterial DNA from fecal samples according to the manufacturing protocol.

#### PCR primer

**Figure 1:**
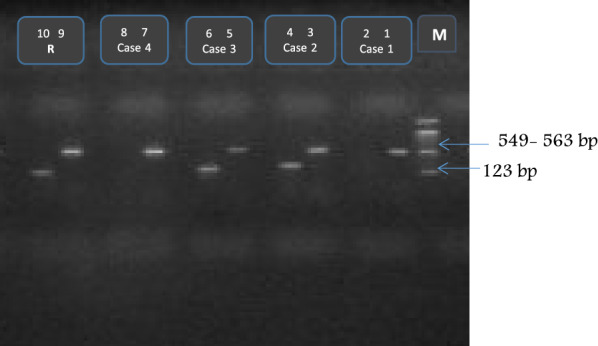
Gel Electrophoresis of Lactobacillus acidophilus and *Bifidobacteria bifidum* gene amplification. Lanes: M, 100 bp DNA ladder marker (Bioron HmbH, Ludwigshafen, Germany). R; reference strains as positive control (*Lactobacillus acidophilus* ATCC 4356, and *Bifidobacterium bifidum* ATCC 11863). Well (1, 3, 5, 7, 9) showed *Lactobacillus acidophilus* gene amplification fragment at 123 bp. Well (4, 6, 10) showed *Bifidobacterium bifidum* gene amplification fragment between 549-563 bp.

Genus specific primers for identification of *Lactobacillus* and *Bifidobacteria* species of lactic acid producing bacteria were designed according to 16S/23S ribosomal RNA intergenic spacer region (16SrRNA) sequence of each species (Sigma, USA) as described by Dubernet et al., and Matsuki et al. [19, 20], respectively. LBA–F (5′-CTT GTA CAC ACC GCC CGT CA-3′), and LBA–R (5-CTC AAA ACT AAA CAA AGT TTC-3) were designed for the amplification of *Lactobacillus* with a PCR product at 123 pb. BIF-R (GGT GTT CTT CCC GAT ATC A), and BIF-F (CTC CTG GAA ACG GGT GG) were designed for the amplification *Bifidobacteria* with a PCR product between 549 and 563 bp (Fig. 1).

#### PCR conditions

The PCR reaction mixture (25 ul) was composed of 1×PCR buffer (50 mM Tris-HCL,PH 8.8, 2.5 mM MgCl2 ,15 Mm (NH4)2SO4 0.45% Triton X-100), 0.2 mM of each dNTP, 25 pmol of each primer, 10 ng of bacterial DNA and 1 U of Taq DNA Polymerase (Biotech International, Australia). The PCR was done in a Touch down Thermal Cycler (Hybaid Middlesex, UK). The PCR amplification program included 1st initial denaturation period for 5 min at 94 °C, followed by 30 cycles involving denaturation at 95 °C for 30 s, annealing at 55 °C for 30 s, extension at 72 °C for 30 s. Finally, the program ended with a final extension step at 72 °C for 7 min. The amplification products were detected using 1% agarose gels (Electrophoresis grade, Invitrogen) electrophoresis. The strain *Bifidobacterium bifidum ATCC 11863* and *Lactobacillus acidophilus ATCC 4356* were used as positive controls for the PCR runs.

### Statistical analysis

Version 12 of the Statistical Program for Social Sciences (SPSS) software were used for data analysis. Mean and standard deviation (SD) or interquartile ranges were used to represent the data. For the comparison between two or three groups, Student’s *t*-test and one-way analysis of variance (ANOVA) with post Hoc analysis (Tukey’s test) were used respectively. For comparing cateviral load and fecalgorical data, Chi-square test was used. Pearson correlation was used for measuring the correlation between the different variables. A P*-*value < 0.05 was considered significant.

## Results

This study enrolled 40 non-cirrhotic chronically infected with hepatitis C virus and 10 healthy control. Clinical and laboratory characteristic were outlined in Table 1**.** There were no statistically significant difference noticed between the patients’ values of liver function tests and the healthy control except in ALT, AST and PT (P-value = 0.003, 0.037 and < 0.001 respectively).

**Table 1:** Clinical and liver profile characteristic of the sample population

	CHC patients (n = 40)	Healthy controls (n = 10)	P-value
Age (years)	43.5 ± 5.97	42.4 ± 7.351	0.621
Gender (M/F ratio) %	28/12 (70%)	6/4 (68%)	0.55
AST (IU/l) ALT (IU/l)	55.000 ± 26.602 58.000 ± 25.631	36.000 ± 16.207 31.400 ± 16.939	0.037* 0.003*
Total bilirubin (mg/dl)	0.761 ± 0.289	0.831 ± 0.103	0.454
Albumin (g/L)	4.275 ± 0.427	4.320 ± 0.235	0.751
Alkaline phosphatase (IU/l)	58.300 ± 14.472	55.600 ± 5.522	0.567
INR PT	1.115 ± 1.108 13.390 ± 0.688	1.060 ± 0.084 12.260 ± 0.881	0.140 < 0.001*

All values were expressed in mean and stander deviation, while gender expressed in number and percentage. CHC; chronic hepatitis c, AST, aspartate aminotransferase; ALT, alanine aminotransferase; INR; international normalized ratio; PT; prothrombin time

^*^ (Significant P-value)

Assessment of HCV viral load revealed that the majority of cases (55%) had high viral load with a median value of 119.403.000 IU/ ml (Table 2).

**Table 2:** Quantitative PCR analysis of HCV viral load among patients’ group

PCR for (HCV) RNA (IU/ ml)		
	N (%)	Median (interquartile)
Mild	10 (25%)	90.800 (18.925, 106.936)
Moderate	8 (20%)	496.143 (301.536, 885.164)
High	22 (55%)	119.403.000 (9160.000, 202000.000)

Data was presented as median and interquartile value

*PCR* polymerase chain reaction, *HCV* hepatitis C virus, *RNA* Ribonucleic acid

On evaluation of hepatic histopathology using Mitavire scoring system, we observed that majority (75%) of the cases (30 out of 40 patients) had a fibrosis score of ≤ 1, and mild activity of disease (A1) was predominant for all HCV cases.

Analysis of gut microbiota in fecal samples showed that a significantly higher number of total lactic acid as well as *Lactobacillus acidophilus* in healthy control compared to chronically infected HCV patients (P value < 0.001) (Tables 3 and 4).

**Table 3:** Comparison between the patients and the control groups as regards total lactic acid bacteria count

GROUPS	Total lactic acid bacterial count/g wet feces weight		P-value
	Range	Median (interquartile)	
CHC patients	0.400–50.000	2.350 (1.200, 7.00)	< 0.001^*^
Healthy controls	22.000–200.000	180.000 (118.000, 192.500)	

*Significant P-value

*CHC* chronic hepatitis c

**Table 4:** Comparison between the patients and the control groups as regards *Lactobacillus acidophilus* count

GROUPS	*Lactobacillus acidophilus* count/g wet feces weight		P-value
	Range	Median (interquartile)	
CHC patients	0.200–20.000	1.000 (0.425, 2.750)	< 0.001^*^
Healthy controls	10.000–80.000	60.000 (40.000, 72.500)	

*CHC* chronic hepatitis c

^*^ Significant P-value

For further confirmation, our genotypic analysis of these intestinal microbiota in the fecal samples revealed that the diversity of the two studied species was higher in the control group compared to the patients group. As detection of the two strains together were lower in the patients group (12 out of 40, 30%) compared to the control one (6 out of 10, 60%). In both patient and control groups, the *Bifidobacteria bifidum* stain was higher (24 out of 40, 60%, and 4 out of 10, 40%) than the *Lactobacillus acidophilus* strain (2 out of 40, 5%, and 0 out of 10, 0%) respectively.

Importantly, when non-cirrhotic HCV patients were stratified according to degree of liver fibrosis, abundance of total lactic acid and *Lactobacillus acidophilus* could be observed higher among those with early stage of fibrosis (score **≤** 1) compared to those with fibrosis score > 1, however, these differences were non-significant ( P = 0.260, P = 0.246 respectively) (Table 5).

**Table 5:** Serum HCV RNA levels, total lactic acid bacterial count, *Lactobacillus acidophilus* count in stool according to liver histopathology result

Group I	Liver histopathology Fibrosis score		P-value
	≤ 1	> 1	
Serum HCV–RNA (IU/ml)	6560.000 (401.000, 119.403.000)	90.800 (18.925, 164.000.000)	0.091
Total lactic acid bacterial Count (CFU/g)	2.750 (1.200, 7.250)	2.000 (0.600, 4.000)	0.260
*Lactobacillus acidophilus* Count (CFU/g)	1.100 (0.475, 3.250)	0.800 (0.250, 1.650)	0.246

All values are presented as median (interquartile range)

*HCV* hepatitis C virus, *RNA* Ribonucleic acid, *CFU* colony forming unit

Furthermore, on assessing the relation between patients’ serum HCV viral load and fecal total lactic acid and *Lactobacillus acidophilus* we noticed negative correlations between the viral load and each of fecal total lactic acid (r = − 0.031, P = 0.850) and fecal *Lactobacillus acidophilus* (r = − 0.005, P = 0.977).

## Discussion

Gut microbiota has recently been known as a main environmental factor persuading the pathogenesis of numerous human diseases [21], comprising hepatic cirrhosis and its relevant complications [22, 23]. Although the relationship between gut microbiota and chronic viral hepatitis has been intensively suggested, this former study investigated the protective role of the total lactic acid gut microbiota (*Lactobacillus acidophilus* and *Bifidobacteria bifidum*) in progression of liver fibrosis in comparison to healthy control. Moreover, numerous studies in the last decades suggested that change in the composition of gut microbiota in cirrhotic patients associated with marked gut dysbiosis led to worsening of the liver disease [16, 24, 25]. However, this contributory role is still an emerging issue. Hence, this study conducted on cases with various serum level of hepatitis C viremia in the early stages of hepatic inflammation before development of severe fibrosis and cirrhosis. Then, the relationships between hepatic histopathological changes and the commonest beneficial gut microbiota namely lactic acid bacteria, *Lactobacillus acidophilus*, and *Bifidobacterium bifidum,* were studied.

In consensus with a published study concerned with the contributory role of lactic acid gut microbiota particularly *Lactobacillus* and *Bifidobacteria* on cases with chronic hepatitis B and cases with decompensated hepatitis B cirrhosis [26], our study showed a significantly lower difference between the count of both the total lactic acid bacteria and *Lactobacillus acidophilus* in patients’ stool samples than normal healthy ones (P < 0.001).

Our results showed that abundant of both fecal total lactic acid bacteria and *Lactobacillus acidophilus* among patients with fibrosis score ≤ 1 compared to those with fibrosis score > 1. However, the fecal count of both of them was statistically insignificant with either the serum HCV viral load (P = 0.850, P = 0.977 respectively) or the score of fibrosis (P = 0.260, P = 0.246 respectively). The non-significant difference may be attributed to the small number of our sample population. In agreement, Jantararussamee et al. demonstrated in an experimental study the hepatoprotective effect of a mixture of lactic acid probiotic bacteria (*L*. *casei*, *L*. *paracasei*, and *W*. *confuse*) in rate under liver fibrosis-inducing conditions. They found that this cocktail diminished the liver oxidative stress, inflammation, and fibrosis [27]. The relationship between altered gut microbiota and hepatic fibrosis appears to be gradual since liver fibrosis was negatively correlated with studied intestinal microbiota.

Moreover, statistical significant differences within the microbial composition (*Lactobacillus acidophilus* and *Bifidobacteria bifidum*) could be observed between our healthy controls and patients chronically infected with HCV. Both *lactobacillus acidophilus* and *Bifidobacteria bifidum* were found to be decreased in patients with HCV. In addition, reduction of gut microbiota was observed with persistent and increased of HCV viral load. Xu et al. investigated the intestinal bifidobacteria composition in 47 subjects, including 16 chronic hepatitis B patients, 16 patients with cirrhotic HBV and 15 normal healthy individuals. Although the results did not reach significant values, *Bifidobacterium longum* was more commonly detected in CHB patients and controls than in HBV cirrhotics (P = 0.011). Thus, the composition of gut *Bifidobacterium* was profoundly changed in virally infected cirrhotic patients (HBV and HCV) with a shift from beneficial species to opportunistic pathogens [28].

Furthermore, our genotypic analysis of gut *Lactobacillus acidophilus ATCC 4356* and *Bifidobacterium bifidum ATCC 11863* microbiota composition in stool samples, revealed that the diversity of these two studied microbiota was higher in healthy control compared to patients chronically infected with hepatitis C virus. In line with this study finding, there were numerous studies which investigated the diversity of various beneficial microbial in cirrhotic chronically infected hepatitis (B or C) patients’ stool [3, 8, 10, 16, 25, 29–32]. In general, low diversity is often associated with diseases, whereas high diversity is seen to be beneficial and is associated with health [15, 33].

The aggravation of the clinical course of patients with chronic liver diseases was suggested to be occurred via gut dysbiosis, as well as, the massive translocation of gut microbiota and its microbial toxic products [34]. It is well known that chronic hepatitis viral infection results in vast translocation of gut microbiota [26, 35]. Such translocation results in impairment of the integrity of gut barrier, which could reach the liver through the portal vein system with subsequent growth of pathogenic bacteria at high rates, as well as, abnormal regulation of immune cells. Hence, severe intestinal inflammation progresses [36]. Furthermore, the loss of gut microbiota homeostasis may worsen the situation during chronic hepatic viral infections due to its great impact on viral replication, in addition to the interactions between the virus and host cells [37, 38].

Beside the immunomodulatory effect of *Lactobacilli* and *Bifidobacteria,* recent studies showed that both of them have antibacterial and antiviral activities [39]. Allam et al. found that the counts of leukocytes, CD3+ T cells and CD56+ natural killer cells were increased after administration of probiotics capsule containing (*L. acidophilus* and *Bifidobacterium* spp.) on a study enrolled on 20 patients with chronic hepatitis C virus infection with enhancement in the HCV treatment response rate of pegylated IFN-α and ribavirin by 25% [39]. A more recent in vitro study (Akter et al.) identified an antimicrobial peptide (AMP) from *Lactobacillus acidophilus* that was antagonistic to Aeromonas hydrophila [40]. Moreover, in another experimental study on a rat model, a mixture of *Bifidobacterium* and various probiotics with galacto-oligosaccharides and fructo-oligosaccharides demonstrated a protective effect against Rotavirus infection. This is mediated by upregulating the expression of TNF-α, IL-4, IFN-γ, and TLR2 promoting their production [41].

Thus, modulation and monitoring of the gut microbiota may represent a new therapeutic avenue for targeted intervention in chronic hepatitis C patients to ameliorate inflammatory process of infection and attenuate the development of fibrosis, hence, to improve the prognosis of the disease. However, further studies, including a larger population sample, as well as, longitudinal studies are required to endorse our findings.

## Limitation of the study

Using culture-based techniques, which are not state of the art, instead of molecular ones is the limitation of our study. However, the technique was used to the best limit of that technology and the study methods were generally well described and reproducible. Regardless, the study may provide marginal value to the body of literature.

## Conclusion

The progression of hepatic fibrosis in chronically infected HCV patients seems to be plausible based on finding the altered *Lactobacillus acidophilus* and *Bifidobacterium bifidum* gut microbiota composition Thus, gut microbiota-targeted interventions may represent a new therapeutic avenue for chronic hepatitis C patients to improve the prognosis of the disease. However, more longitudinal studies in addition to a larger population sample, are necessary to authorize our findings

## Data Availability

All relevant data are within the paper.

## Abbreviations

HCV: Chronic hepatitis C
LPS/TLR4: Lipopolysaccharide/ toll-like receptor 4
CDR: Cirrhosis dysbiosis ratio
HBs Ag: Hepatitis B surface antigen
HIV Ab: Human immune deficiency antibody
qPCR: Real-time polymerase chain reaction
